# Supplementary material for: The aberrant upregulation of exon 10-inclusive SREK1 through SRSF10 acts as an oncogenic driver in human hepatocellular carcinoma
Source: Nat Commun. 2022 Mar 16;13:1363. doi: 10.1038/s41467-022-29016-x (PMC8927159; doi:10.1038/s41467-022-29016-x)
Supplement: Supplementary file 1 — Supplementary Information [file 41467_2022_29016_MOESM1_ESM.pdf]

Supplementary information

The aberrant upregulation of exon 10-inclusive SREK1 through SRSF10 acts as an oncogenic driver in human hepatocellular carcinoma

Cunjie Chang[1,2#], Muthukumar Rajasekaran[3#], Yiting Qiao[4#], Heng Dong[1,2#], Yu Wang[3], Hongping Xia[3], Amudha Deivasigamani[3], Minjie Wu[1], Karthik Sekar[3], Hengjun Gao[5], Mengqing Sun[1], Yuqin Niu[6], Qian Li[1], Lin Tao[1,2], Zhen Yan[1], Menglan Wang[1], Shasha Chen[7], Shujuan Zhao[1,2], Dajing Chen[1,2], Lina Li[1], Fan Yang[1], Haojin Gao[1], Baodong Chen[1], Ling Su[1], Liang Xu[8], Ye Chen[8], Veerabrahma Pratap Seshachalam[3], Gongxing Chen[1,2], Jayantha Gunaratne[9], Wanjin Hong[9], Junping Shi[10], Gongying Chen[11], David S. Grierson[12], Benoit Chabot[12], Tian Xie[1,2*], Kam Man Hui[1,3,9,13*], Jianxiang Chen[1,2,3*]

Supplementary Table 1 Summary of the clinic pathological characteristics of the 10 HCC patients employed for the RT-PCR in Figure 1C.

**Group**	**SREK1$^{L/}$ SREK1^{S} (T/MN)>2 (*n*=5)**	**SREK1$^{L/}$ SREK1^{S} (T/MN)<2 (*n*=5)**
**Gender**		
Male	5	5
Female	0	0
**Age**		
≤60	5	3
>60	0	2
**AFP**		
≤20 ng	1	5
>20ng	4	0
**Cirrhosis status**		
Yes	3	3
No	2	2
**Tumor size**		
≤5cm	1	4
>5cm	4	1
**TNM Stage**		
I	4	3
II & Above	1	2
**BCLC Stage**		
A	4	3
B&C	1	2

Supplementary Table 2 Summary of clinic pathological characteristics of the 60 HCC patients employed in this study

Group	$SREK1^{L}/SREK1^{S}$ Low (*n*=30)	$SREK1^{L}/SREK1^{S}$ High (*n*=30)
**Gender**		
Male	27	26
Female	3	4
**Age**		
≤60	21	24
>60	9	6
**Cirrhosis status**		
Yes	20	17
No	10	13
**Tumor size**		
≤5cm	16	20
>5cm	14	10
**AFP Level**		
≤20 ng/ml	12	15
>20 ng/ml	18	15
**Recurrence Status**		
R (≤ 2-years)	13	20
NR (> 2-years)	16	11

Supplementary Table 3 Clinic pathological characteristics of the 48 HCC patients employed for the SREK1^{L} IHC assay

	**IHC low expression (*n*=27)**	**IHC high expression (*n*=21)**
**Gender**		
Male	25	18
Female	2	3
**Age**		
≤60	23	16
>60	4	5
**Tumor size**		
≤5cm	14	8
>5cm	13	13
**Cirrhosis status**		
Yes	17	17
No	10	4
**Recurrence**		
Yes	9	16
No	18	5
**TNM Stage**		
I	20	13
II	3	4
III A,B&C	4	4
**BCLC Stage**		
0 & I	22	16
II	4	3
IIIA	3	2
**HBs Antigen**		
Yes	24	17
No	3	4

The expression was scored from 1-4 and the average scores were used for analysis. Scores ≤ 2.0 = low expression; scores > 2.0 = high expression.

Supplementary Table 4 Clinic pathological correlation analysis of the HCC patients with the SRSF10 gene expression.

	SRSF10 low expression (*n*=35)	SRSF10 high expression (*n*=35)	x^2 or fisher exact test	P value
**Gender**				
Male	33	28	3.188	0.07
Female	2	7		
**Age**				
<50	4	10	3.214	0.07
≥50	31	25		
**AFP**				
Negative	22	12	5.719	**0.01**
Positive	13	23		
**Differentiation**				
I/II	24	23	0.065	0.7
III/IV	11	12		
**Size**				
≤5 cm	21	18	0.521	0.4
>5 cm	14	17		
**Child-Pugh score**				
A	26	27	0.078	0.7
B	9	8		
**Cirrhosis**				
No	18	14	0.921	0.33
Yes	17	21		
**Venous Infiltration**				
NA	25	19	2.2	0.1
VI	10	16		
**Recurrence**				
≤24 months	17	24	2.57	0.109
≥24 months	18	11		
**Status**				
Dead	14	23	4.644	**0.03**
Alive	21	12		

Supplementary Table 5 List of the antibodies used in the study.

Antibodies used for Western blot					
**Cat. No**	**Antibody**	**Lot No.**	**Species**	**dilution**	**Company and description**
HPA037674	SREK1	R35896	Rabbit	1/1000	Sigma-Aldrich, for Fig. 5G, Fig. 3E (ab-2)
#2118	GAPDH (14C10)	10	Rabbit	1/5000	Cell Signaling Technology
T6199	α-Tubulin (DM1A)	R11234	Mouse	1/5000	Sigma-Aldrich
sc-393594	UPF1	L1217	Mouse	1/2000	Santa Cruz Biotechnology
sc-374557	SMG1	K2817	Mouse	1/1000	Santa Cruz Biotechnology
sc-515722	MOV10	H0916	Mouse	1/1000	Santa Cruz Biotechnology
sc-393905	RUVBL1	H0217	Mouse	1/1000	Santa Cruz Biotechnology
sc-374135	RUVBL2	K2817	Mouse	1/1000	Santa Cruz Biotechnology
sc-374230	UPF2	H1417	Mouse	1/1000	Santa Cruz Biotechnology
sc-398821	UPF3	G3117	Mouse	1/1000	Santa Cruz Biotechnology
sc-271405	MAGOH/B	G0210	Mouse	1/1000	Santa Cruz Biotechnology
sc-514050	GNL2	I1217	Mouse	1/1000	Santa Cruz Biotechnology
sc-514308	SEC13	G2617	Mouse	1/1000	Santa Cruz Biotechnology
sc-32318	PABPC1	L2216	Mouse	1/1000	Santa Cruz Biotechnology
HPA053805	SRSF10	R71755	Rabbit	1/1000	Sigma-Aldrich
LS-C133661	SRSF10	NA	Mouse	1/2000	LSBio
sc-33652	SRSF1	L0413	Rabbit	1/1000	Santa Cruz Biotechnology
HPA021593	PECR	R09325	Mouse	1/1000	Santa Cruz Biotechnology
Sc-5261	ELAVL1/HuR	NA	Mouse	1/1000	Santa Cruz Biotechnology
Sc-101137	SFRQ	NA	Mouse	1/1000	Santa Cruz Biotechnology
HPA034829	SF3B6	R32766	Mouse	1/1000	Sigma-Aldrich
NA	$SREK1^{L}$	NA	Rabbit	1/5000	Customized made by Genscript (ab-1 of Fig 3E)
ab21679	CHC/CLTC	GR264025-2	Rabbit	1/5000	Abcam
ab237697	TXNDC5	NA	Rabbit	1/1000	Abcam
ab175205	BLOC1S5	NA	Rabbit	1/2000	Abcam
A305105A	$SREK1^{L}$ and $SREK1^{S}$	1	Rabbit	1/5000	Thermofisher
sc-374015	Lamin B1	J2220	Mouse	1/5000	Santa Cruz Biotechnology

Antibodies used for IP and SILAC					
**Cat. No**	**Antibody**	**Lot No.**	**Species**	**Company**	**Application**
HPA037674	SREK1	R35896	Rabbit	Sigma-Aldrich	IP
A302-282A-1	SRSF10	NA	Rabbit	Bethyl Bio	IP for SILAC
HPA053805	SRSF10	R71755	Rabbit	Sigma-Aldrich	IP for SILAC

NA	SREK1-E10	NA	Rabbit	Customized, made by Genscript	IP
I5006	IgG control	NA	Rabbit	Sigma-Aldrich	IP control
ab99617	Rat monoclonal [187.1] Secondary Antibody to Mouse kappa - light chain (HRP)	GR1190308-3	Rat	Abcam	IB, 1/5000
ab99697	Mouse monoclonal [SB62a] Secondary Antibody to Rabbit IgG light chain (HRP)	GR122803-1	Mouse	Abcam	IB, 1/5000

Antibodies used for IF

Cat. No	Antibody	Lot No.	Species	Company	Dilution
ab89984	α-Tubulin	GR271216-4	Chicken	Abcam	1/1000
F9291	FLAG® BioM2/Flag-Biotin	SLBF5390V	Mouse	Sigma-Aldrich	1/500
HPA037674	SREK1	R35896	Rabbit	Sigma-Aldrich	1/200
LS-C133661	SRSF10	NA	Mouse	LSBio	1/100
ab5176	Anti-Histone H3 (phospho S10)	NA	Rabbit	Abcam	1/200

Supplementary Table 6 Primers for real-time PCR.

Primer Name	Sequence 5'-3'
ACTB F	AATCTGGCACCACACCTTCTA
ACTB R	ATAGCACAGCCTGGATAGCAA
GAPDH F	ATGGGGAAGGTGAAGGTCG
GAPDH R	GGGGTCATTGATGGCAACAATA
18S F	GTAACCCGTTGAACCCCATT
18S R	CCATCCAATCGGTAGTAGCG
SREK1 E9E10J-F	GCATTCACGGGACAAGAGAAAAG
SREK1 E10E11J-R	CCTTTCCCTGCTGGAACTACGA
SREK1 E9-F	GAGTAGATCCCATAATAG
SREK1 E9E11J-R	TACGCCTTTCCCGTGAAT
SREK1 E9-F	CGTTCAAGACAGAAAGACAGAC
SREK1 E11-R	AACTCCTGCTCCTCCTCCTA
SRSF10 F	CGACAATGATAGCCAAGTAAG
SRSF10 R	TCTATGTAGCACCTTTCCTC
SRSF1 F	GCAGGTGATGTATGTTATGC
SRSF1 R	TCTCCCTCATGAGATCTAAAC
ELAVL1 F	GATCAGACTACAGGTTTGTC
ELAVL1 R	TTGAAACTGGTAATTGCCTC
MAGOH F	CCAAAGAGGATGATGCATTG
MAGOH R	CTGGATCCTTGGATTGATTG
P*OLR2D* F	AAAAACAGAGAGACCATTGC
POLR2D R	TATCATCAAGAATCTGCTGC
POLR2G F	CATGTCTTGCTTCATCTCTC
POLR2G R	CCTCATCCATTGTCTTGTAAC
SFPQ F	CTTGAATCTAGAGCTTTGGC
SFPQ R	CACTATTACAACAGCCCTTTC
PABPC4 F	GCACAAAAGAAAGTAGAACG
PABPC4 R	CCTGATATCGACTAATTCTCTC
PABPC1 F	AATGAACGGTAGAATTGTGG
PABPC1 R	GTTCTGAGTCTGTGGGATAG
MAGOHB F	TCAGTCAAAGGATCCTGAAG
MAGOHB R	CAAACAGCCTGAAAACATAC
PECR F	GTACCAGATCATGACAACTG
PECR R	CAGAAGCATATCCTCAAGATG
BLOC1S5-TXNDC5-F	ACGAAAAAAGGTGTGGACAC

BLOC1S5-TXNDC5-R	ATACAGCCCTTGCTTGAGC
ABHD14A-ACY1-F	TGACCTTCCAGCTCACCACG
ABHD14A-ACY1-R	TGGATGCTGACGCACTTCA
SYS1-DBNDD2-F	CCCTCACCTGGAGCTGACAT
SYS1-DBNDD2-R1	TCGCAAGGCAAGAACACA
TXNDC5-F	CGTGGTCTTCGAGAAATGCG
TXNDC5-R	AGGTCATTCCAAGTCGGCTG3
BLOC1S5-F	GCTGTGTGTTCTGACGGTG
BLOC1S5-R	CTGTCCCGCATTGTGTCTCTA
UPF1-F	ACCGACTTTACTCTTCCTAGCC
UPF1-R	AGGTCCTTCGTGTAATAGGTGTC
Mouse $Srek^{L}$-F	TCACGGGACAAGAGAAAAG
Mouse $Srek1^{L}$-R	CCTTTCCCTGCTGGTACTACGA

Supplementary Figures and figures Legends

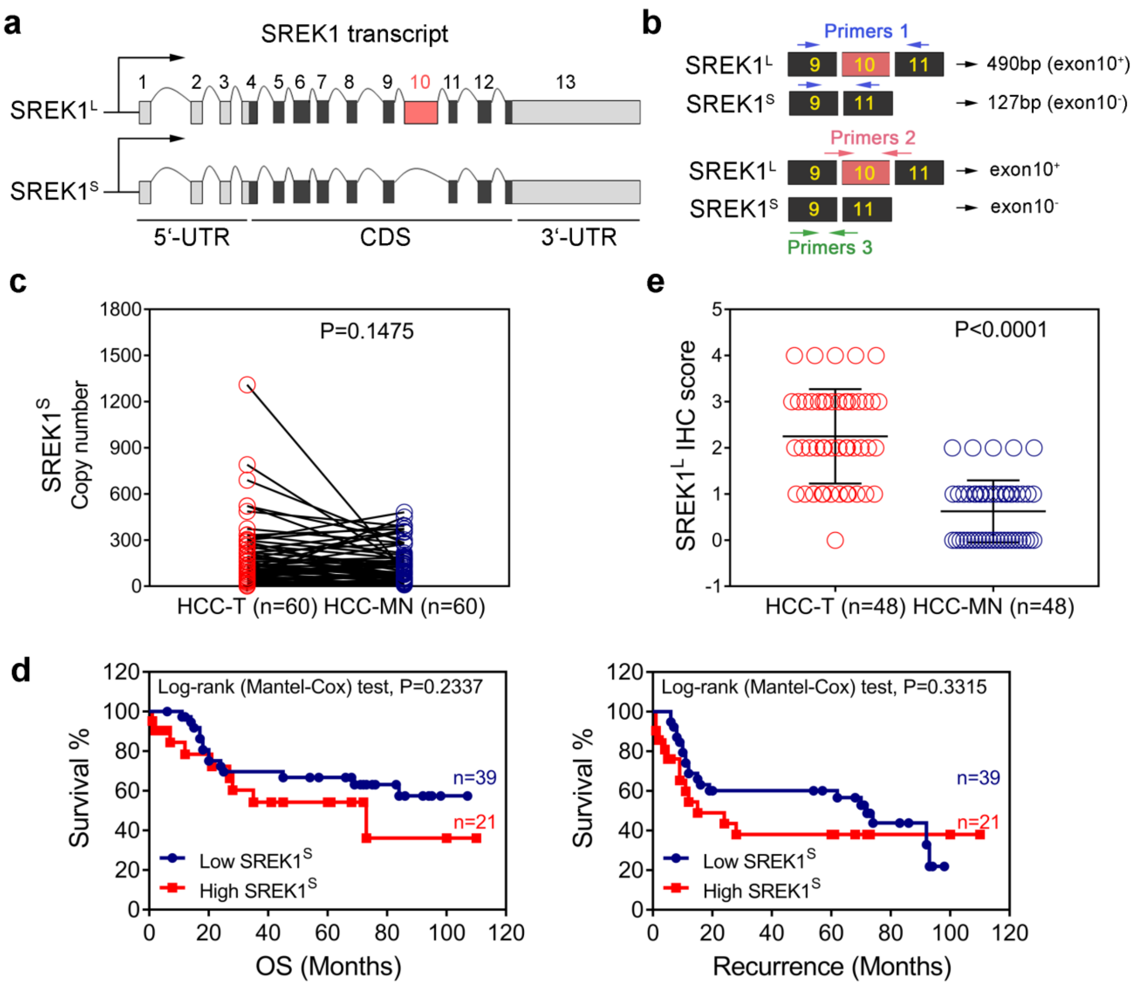

Supplementary Fig. 1 SREK1^{L}, but not SREK1^{S}, is up-regulated in HCC tumors and associated with HCC prognosis. (**a**) Two SREK1 variants: with (SREK1^{L}) or without (SREK1^{S}) exon 10. (**b**) Three sets of primers (primer sets 1-3) were designed to detect the two variants. (**c**) The real-time PCR detection of SREK1^{S} expression in 60 pairs of HCC tissues (HCC-T: HCC tumors; HCC-MN: HCC matched adjacent histologically normal tissues. (**d**) The survival analysis of high and low expression of SREK1^{S} cutoff by mean in 60 patients' tissues. (**e**) Summary of the results obtained from immunohistochemical staining of SREK1^{L} by an SREK1-E10 antibody in 48 pairs HCC tissues. Data are shown as the mean ± SD, two-tailed, paired *t* test is used for (**c**, **e**), ∗∗p <0.01; ∗∗∗p <0.001. Source data are provided as a Source Data file.

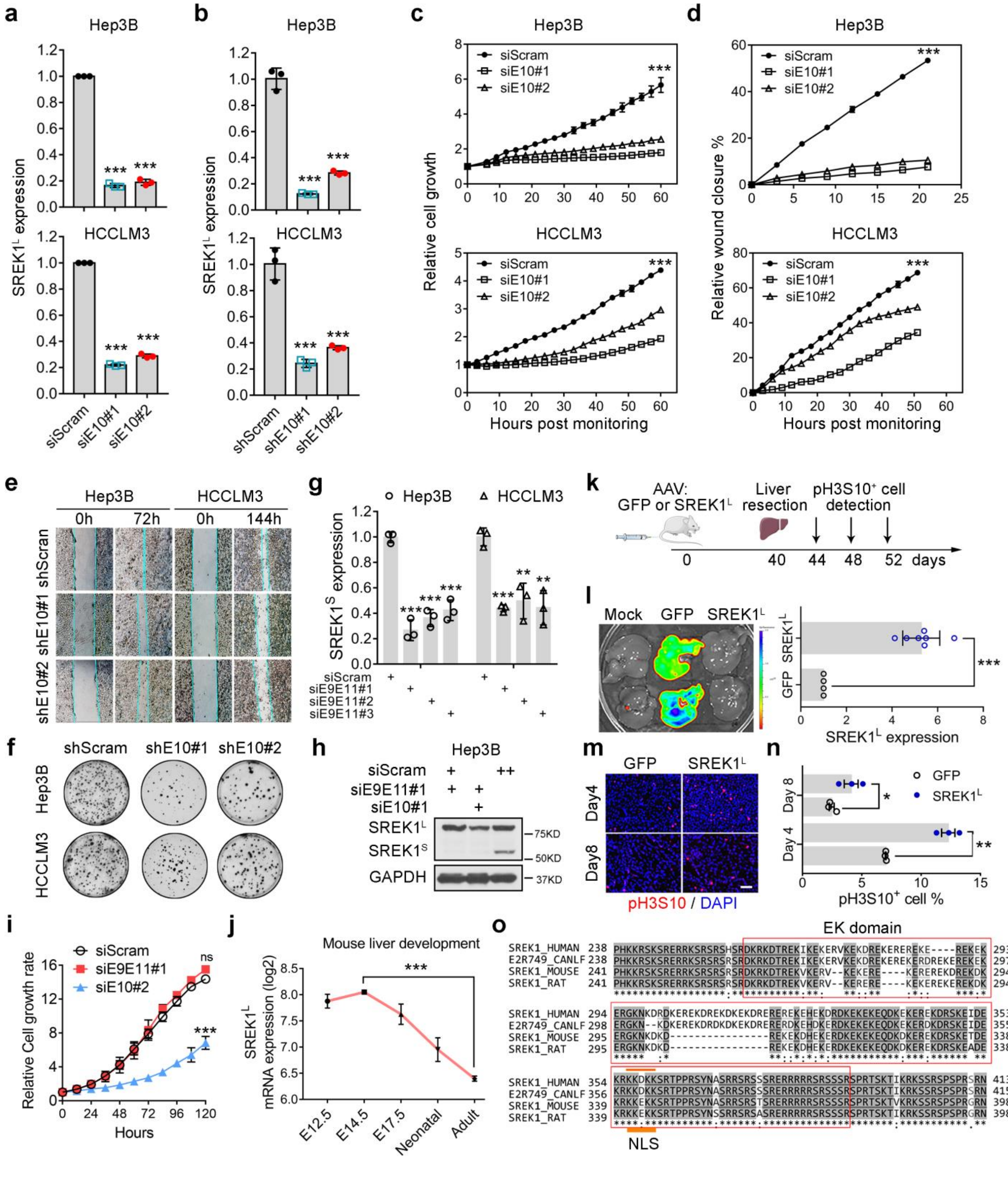

Supplementary Fig. 2 Depletion of SREK1^{L} inhibits proliferation and migration of HCC cells. (**a**) The knockdown of SREK1^{L} by two siRNA targeting exon10-coded mRNA in two HCC cell lines by real-time PCR (*n*=3, data are shown as the mean ± SD; ***p <0.001). (**b**) The mRNA expression of SREK1^{L} in Hep3B or

HCCLM3 stable knockdown cells (n=3, data are shown as the mean ± SD; $^{***}p$ <0.001). ShScram: scramble control shRNA, shE10#1 and #2: exon 10-specific shRNAs. (**c**) The cell growth, and (**d**) Wound healing migration were analyzed in scramble- or SREK1^{L}-knockdown cells (1% FBS was used for wound healing assay to exclude the potential growth effect on migration) (n=3, data are shown as the mean ± SEM; $^{***}p$ <0.001). (**e**) Wound healing migration assays or (**f**) anchorage-independent soft agar colony formation assays of cells in which exon 10-specific SREK1 was stably knocked down. (**g**) PCR detection of the SREK1^{S} expression in cells treated by three individual siRNAs targeting SREK1^{S} (n=3, data are shown as the mean ± SEM; $^{**}p$<0.01, $^{***}p$ <0.001). (**h**) Immunoblotting analysis of the SREK1^{L} and SREK1^{S} protein expression in the Hep3B cells treated by the siRNAs targeting SREK1^{S} or SREK1^{S} combined with SREK1^{L}. (**i**) Incucyte analysis of the Hep3B cell growth after siRNA treatment (n=3, data are shown as the mean ± SD; $^{***}p$ <0.001). (**j**) Expression of SREK1 during mouse liver development (n=3, data are shown as the mean ± SD; $^{***}p$ <0.001). (**k**) The schedule of Adeno-associated Virus (AAV) in vivo delivery of GFP and Srek1^{L} expression by intravenous injection, partially hepatectomy (30% removed) was performed on day 40 and the pH3S10^{+} cells were detected on day 44, 48 and 52. (**l**) The GFP (n=4) or Srek1^{L} (n=7) expression was detected by small animal imaging (left panel) or realtime PCR (right panel) (data are shown as the mean ± SD; $^{*}p$ <0.05, $^{**}p$ <0.01), and pH3S10^{+} cells were detected by (**m**) immunofluerenscence staining and were quantified (**n**) on the day 4th and 8th after hepatectomy (n=3, data are shown as the mean ± SD; $^{***}p$ <0.001), scale bar = 100 µm. (**o**) The alignment of the consensus protein sequences for SREK1 EK domain in four species and the NLS signal is labelled with an orange line. Two-tailed, unpaired *t* test is used for (**a-d**, **g**, **i**, **j**, **l**, **n**). Source data are provided as a Source Data file. Images in Supplementary Fig. 2**k** provided with permission from Servier Medical Art.

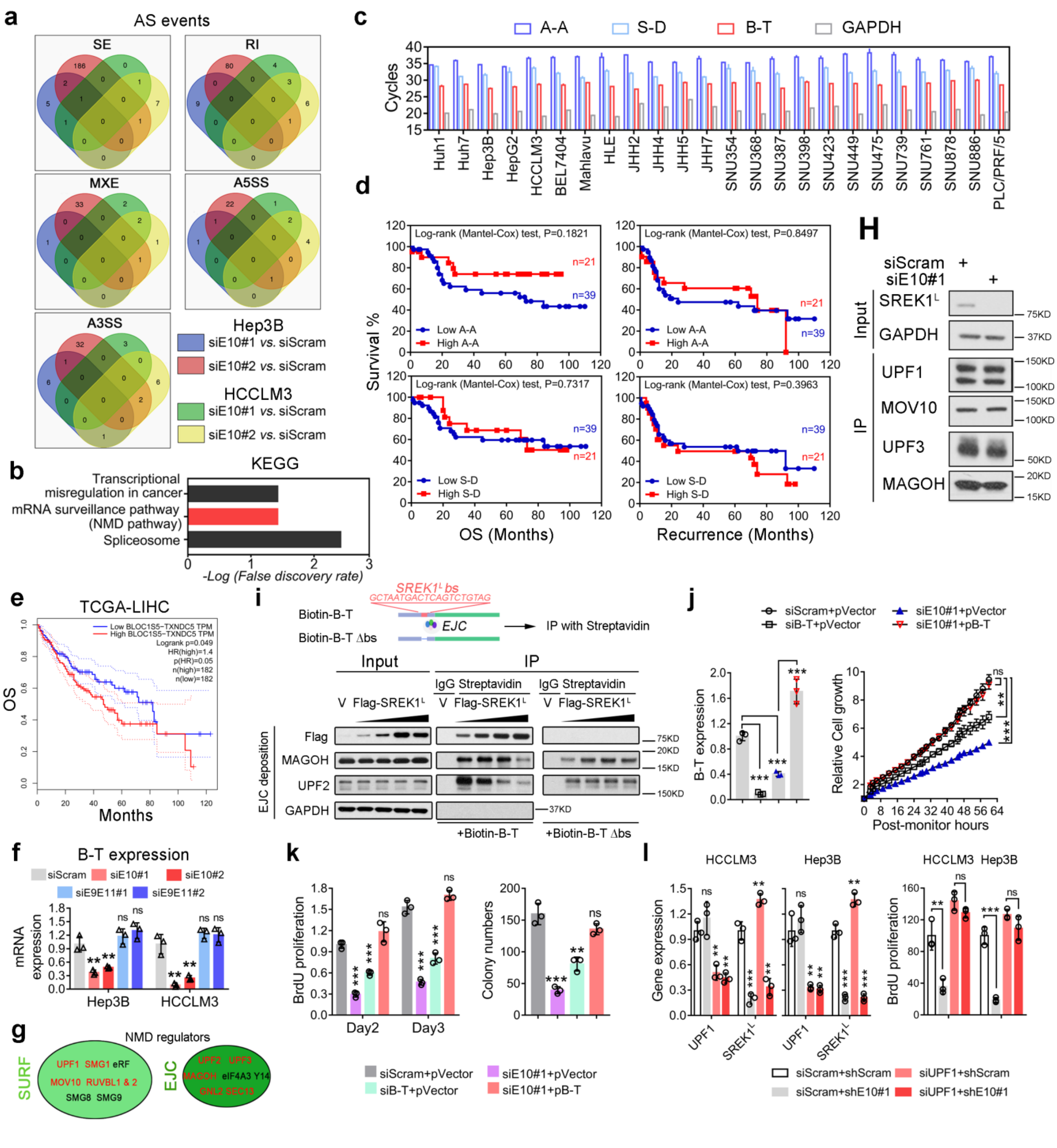

Supplementary Fig. 3 SREK1^{L} maintains the expression of B-T, a NMD target gene, to promote the oncogenesis of HCC cells. (**a**) Venn diagram analysis of the targets regulated by the 5 common AS of SREK1^{L} in Hep3B and HCCLM3 cells. SE: Skipped Exon, RI: Retained Intron, MXE: Mutually exclusive

Exon, A5SS: Alternative to 5′Splice Site, and A3SS: Alternative to 3′Splice Site. (**b**) The KEGG pathway enrichment by the top 100 SREK1-coexpressed genes in TCGA-LIHC database. (**c**) The analysis of the cycles for real time PCR on three NMD target genes and GAPDH expression in 24 HCC cell lines. (**d**) Comparison of the OS and Disease –free survival of high or low expression of ABHD14A-ACY1 (A-A) and SYS1-DBNDD2 (S-D) in 60 HCC patients. (**e**) The OS analysis of the high or low B-T expression in TCGA-LIHC database. (**f**) The expression of B-T in scramble (siScram)-transfected, $SREK1^{S}$ (siE9E11#1 or 2) or $SREK1^{L}$ (siE10#1 or 2)-knockdown Hep3B and HCCLM3 cells (*n*=3, data are shown as the mean ± SD; **p <0.01). (**g**) The components of two reported key NMD complexes—the SURF complex and EJC; elements chosen for detection are labelled in red. (**h**) The immunoblotting analysis of the corresponding targeted protein expression in the input or immunoprecipitation for RIP assay shown in Fig. 3**f-g** in Hep3B cells. (**i**) Western blots show Flag-tagged $SREK1^{L}$ and endogenous EJC components MAGOH and UPF2 coprecipitated with biotinylated B-T or B-T ΔBS ($SREK1^{L}$ binding site deleted) RNA in Hep3B nuclear extract. (**j**) Real-time PCR detection of the B-T expression (*n*=3, data are shown as the mean ± SD; ***p <0.001) and the growth curve (*n*=3, data are shown as the mean ± SEM; **p <0.01, ***p <0.001) are shown for different treatment groups in Hep3B cells. (**k**) The BrdU proliferation or anchorage-independent soft agar colony formation assays were performed in scram control, $SREK1^{L}$, B-T knockdowns or $SREK1^{L}$ knockdown combined with B-T re-expression in Hep3B cells (*n*=3, data are shown as the mean ± SD; **p <0.01, ***p <0.001). (**l**) Real-time PCR detection of the gene expression and the BrdU proliferation analysis in the Hep3B and HCCL3M cells silenced with scramble, UPF1, $SREK1^{L}$ or their combined siRNAs (*n*=3, data are shown as the mean ± SD; **p <0.01, ***p <0.001). Two-tailed, unpaired *t* test is used for (**f**, **j**, **k**, **l**). Source data are provided as a Source Data file.

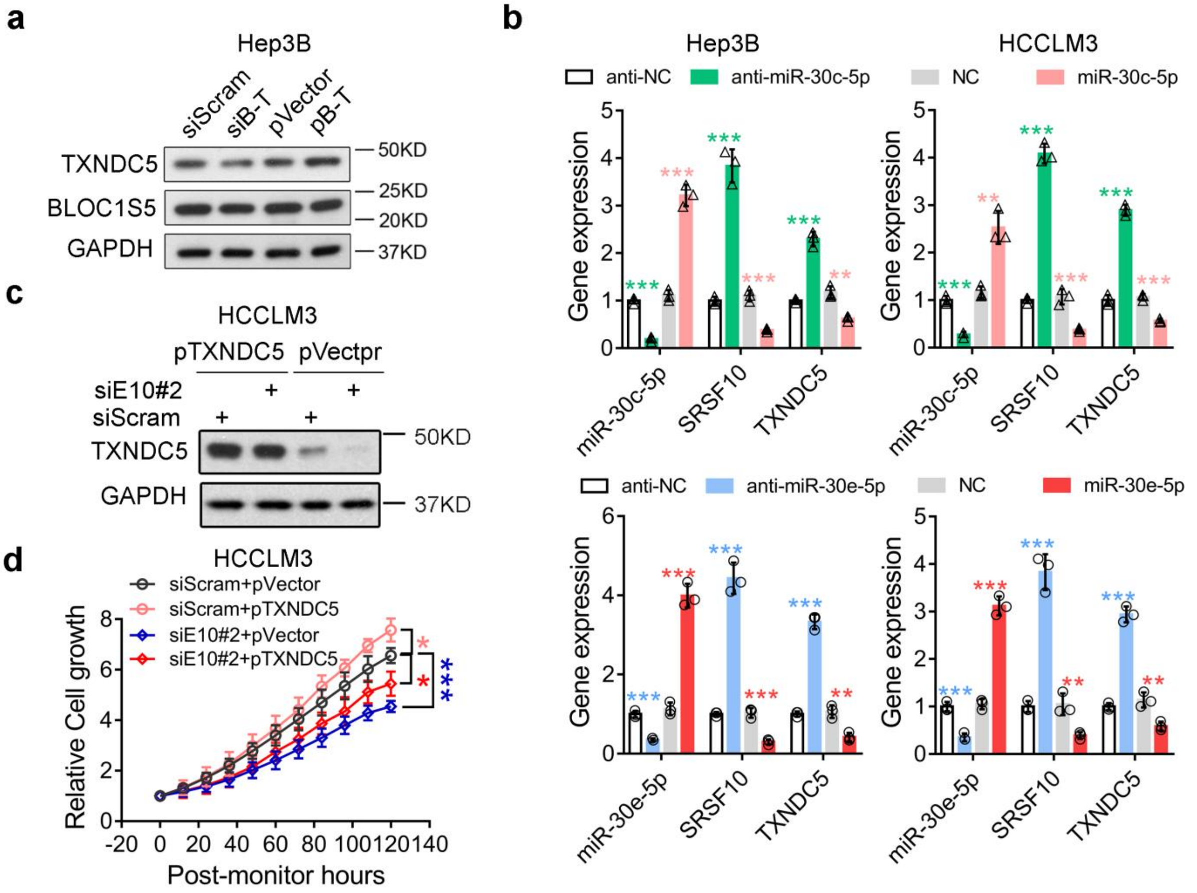

Supplementary Fig. 4 B-T acts as a ceRNA to inhibit miR-30c-5p and miR-30e-5p and promote SRSF10 and TXNDC5 expression in HCC cells. (**a**) The immunoblotting analysis of the effect of knockdown or forced expression of B-T on TXNDC5 and BLOC1S5 protein expression in Hep3B cells. (**b**) Realtime PCR analysis of the effect of the inhibitor or mimics of miR-30c-5p or miR-30e-5p on the endogenous expression of TXNDC5 or SRSF10 genes in Hep3B and HCCLM3 cells (*n*=3, data are shown as the mean ± SD; **p <0.01, ***p <0.001). (**c**) Immunoblotting analysis of TXNDC5 and GAPDH expression after SREK1^{L} or scramble control knockdown in the TXDNC5 or vector control overexpressed HCCLM3 cells. (**d**) Incucyte cell growth assay of the TXDNC5 or vector control overexpressed HCCLM3 cells after scramble or SREK1^{L} knockdown

treatment (*n*=3, data are shown as the mean ± SEM; *p <0.05, ***p <0.001). Two-tailed, unpaired *t* test is used for (**b**, **d**). Source data are provided as a Source Data file.

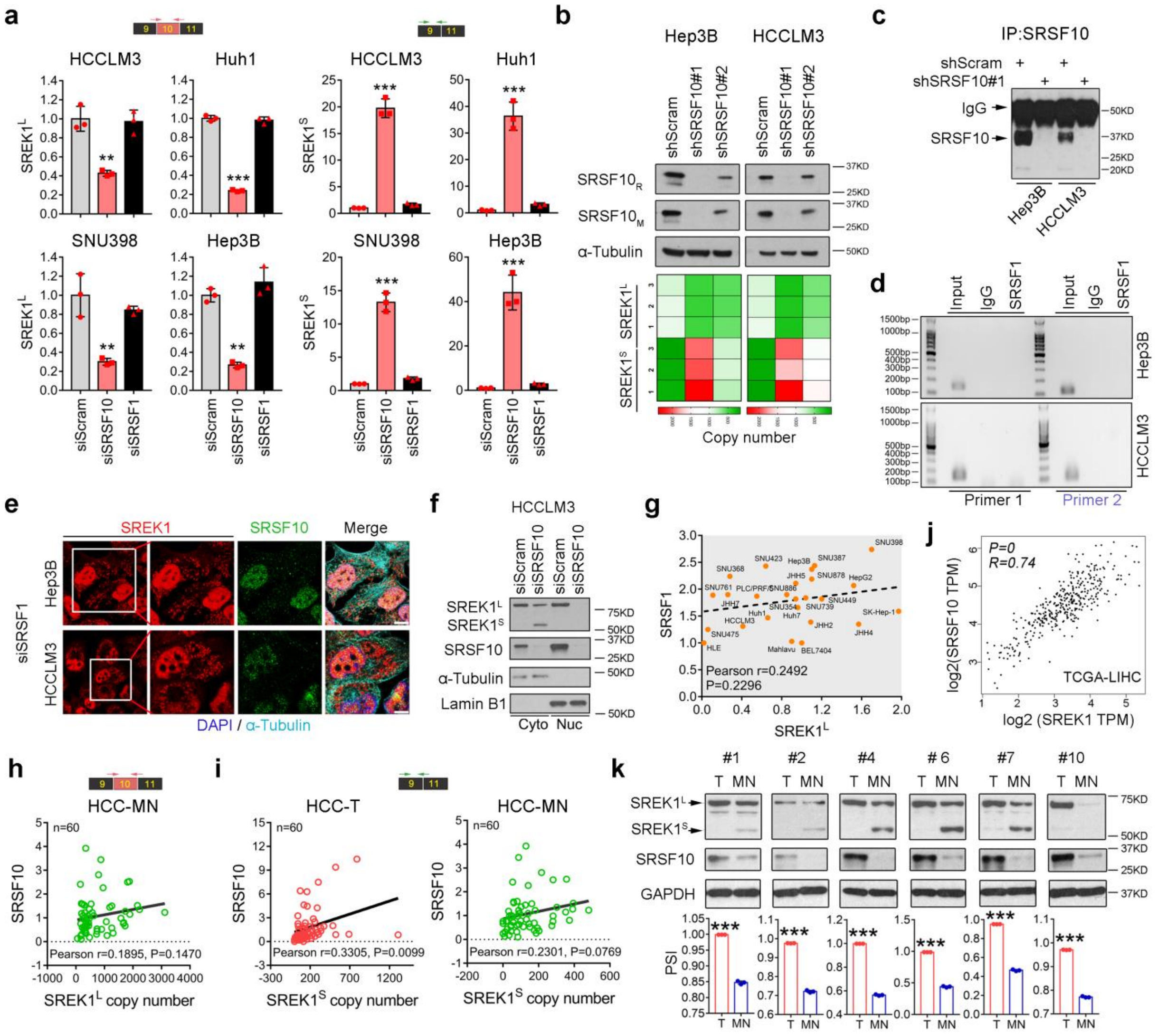

Supplementary Fig. 5 SRSF10, but not SRSF1, regulates exon10 splicing of SREK1. (**a**) Analysis of the relative mRNA expression of SREK1^L or SREK1^S after knockdown of scramble, SRSF10 or SRSF1 in four HCC cell lines by real-time PCR assay (*n*=3, data are shown as the mean ± SD; **p <0.01, ***p <0.001). (**b**) Western blot (upper panel) and heatmap showing the efficiency of mRNA expression (lower panel), with data shown as copy numbers, following stable SRSF10 knockdown in Hep3B and HCCLM3 cells. (**c**) Immunoprecipitation of endogenous SRSF10 in stable shSRSF10#1- or shScram-transfected Hep3B and HCCLM3 cells using a commercially available antibody against SRSF10. (**d**) DNA gel detection of SREK1^L binding in the RIP by SRSF1 or IgG in Hep3B or HCCLM3 cells. (**e**) The immunofluorescence confocal

microscopy analysis of the endogenous SREK1 (red), SRSF10 (green) and α-Tubulin (fake color) in SRSF1-knockdown Hep3B and HCCLM3 cells, scale bar = 5 μm. (**f**) Nuclear and cytoplasmic extraction to detect two SREK1 variants protein expression after SRSF10 or scramble control knockdown in HCCLM3 cells, cyto: Cytoplasm, Nuc: nucleus. (**g**) Correlation analysis of the expression of $SREK1^L$ with SRSF1 protein expression in 25 HCC cell lines. *Pearson* correlation analysis of the expression of SRSF10 with (**h**) $SREK1^L$ in 60 HCC match normal (HCC-MN), (**i**) $SREK1^S$ in 60 HCC-T or HCC-MN tissues, or (**j**) $SREK1^L$ in TCGA-LIHC database. (**k**) Immunoblotting analysis of $SREK1^L$, $SREK1^S$ and SRSF10 protein expression in six pair HCC tissues, and the PSI (Percentage-splice-in = splice_in / (splice_in+splice_out)) was calculated based on the quantification of two variants (n=3, data are shown as the mean ± SD; ***p <0.001). Two-tailed, unpaired t test is used for (**a**, **k**).Source data are provided as a Source Data file.

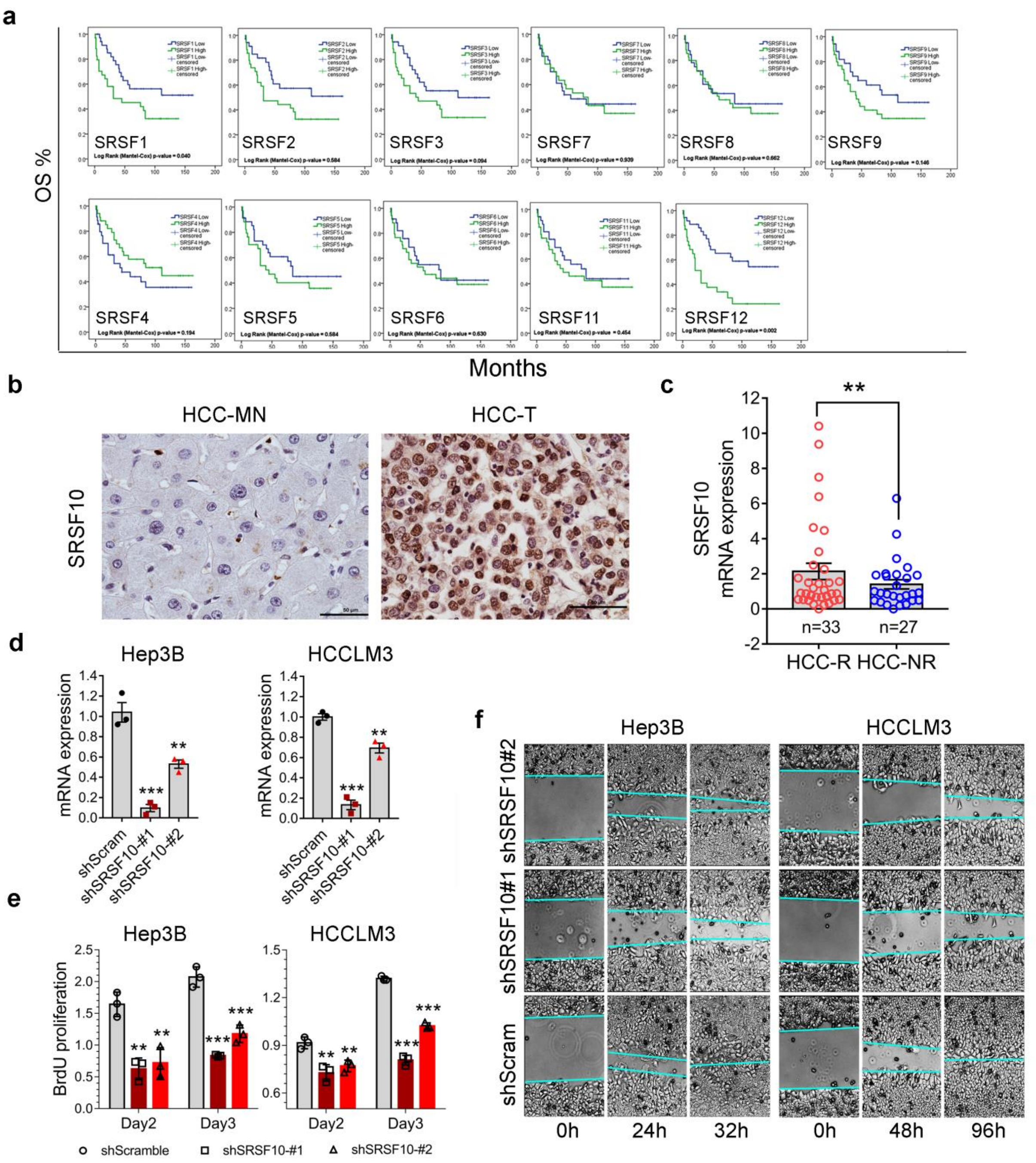

Supplementary Fig. 6 SRSF10 is associated with the prognosis of HCC patients, and acts as an oncogenic driver in HCC. (**a**) Comparison of the OS of the high and low expression (using medium as the cut-off) of the rest 11 SRSF factors based on our microarray dataset information. (**b**) Representative IHC images of SRSF10 expression in matched normal (MN) and tumor (T) HCC tissues. (**c**) SRSF10 gene

expression significantly correlated with the recurrence of the HCC patients based on the analysis of 60 pairs of HCC tissues cohort by real time PCR assays (data are shown as the mean ± SD; $^{**}p < 0.01$). HCC-R: HCC recurrence; HCC-NR: HCC non-recurrence. (**d**) Real time PCR analysis of the expression of stable knockdown of SRSF10 or scramble in Hep3B and HCCLM3 cells (*n*=3, data are shown as the mean ± SD; $^{**}p < 0.01$, $^{***}p < 0.001$). (**e**) BrdU proliferation (*n*=3, data are shown as the mean ± SD; $^{**}p < 0.01$, $^{***}p < 0.001$) and (**f**) the wound healing assays of the stable knockdown of SRSF10 or scramble in Hep3B and HCCLM3 cells. Two-tailed, unpaired *t* test is used for (**c**, **d**, **e**). Source data are provided as a Source Data file.

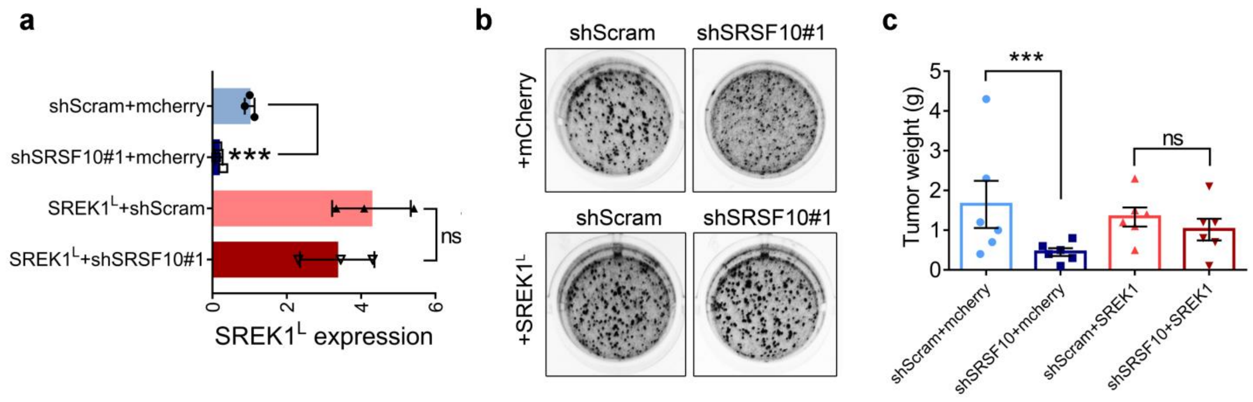

Supplementary Fig. 7 SREK1^{L} is an oncogenic downstream effector of SRSF10 to promote the carcinogenesis of HCC cells. (**a**) SREK1^{L} expression in various stable HCCLM3 cells as indicated (*n*=3, data are shown as the mean ± SD; ***p <0.001). (**b**) Anchorage-independent soft agar colony formation assays of stable knockdown of SRSF10 or the combination of the forced expression of SREK1^{L} in HCCLM3 cells. (**c**) The xenograft tumor weight obtained following injection with the various stable HCCLM3 cells in mouse xenograft tumourigenesis experiments (*n*=6, data are shown as the mean ± SEM; ***p <0.001). Two-tailed, unpaired *t* test is used for (**a**, **c**). Source data are provided as a Source Data file.
