# Supplementary material for: The aberrant upregulation of exon 10-inclusive SREK1 through SRSF10 acts as an oncogenic driver in human hepatocellular carcinoma
Source: Nat Commun. 2022 Mar 16;13:1363. doi: 10.1038/s41467-022-29016-x (PMC8927159; doi:10.1038/s41467-022-29016-x)
Supplement: Supplementary file 3 — Reporting Summary [file 41467_2022_29016_MOESM3_ESM.pdf]

nature portfolio

Corresponding author(s): Tian Xie, Kam Man Hui, Jianxiang Chen

Last updated by author(s): Feb 8, 2022

Reporting Summary

Nature Portfolio wishes to improve the reproducibility of the work that we publish. This form provides structure for consistency and transparency in reporting. For further information on Nature Portfolio policies, see our Editorial Policies and the Editorial Policy Checklist.

Statistics

For all statistical analyses, confirm that the following items are present in the figure legend, table legend, main text, or Methods section.

n/a	Confirmed	
☐	☒	The exact sample size (*n*) for each experimental group/condition, given as a discrete number and unit of measurement
☐	☒	A statement on whether measurements were taken from distinct samples or whether the same sample was measured repeatedly
☐	☒	The statistical test(s) used AND whether they are one- or two-sided<br>*Only common tests should be described solely by name; describe more complex techniques in the Methods section.*
☒	☐	A description of all covariates tested
☒	☐	A description of any assumptions or corrections, such as tests of normality and adjustment for multiple comparisons
☐	☒	A full description of the statistical parameters including central tendency (e.g. means) or other basic estimates (e.g. regression coefficient) AND variation (e.g. standard deviation) or associated estimates of uncertainty (e.g. confidence intervals)
☐	☒	For null hypothesis testing, the test statistic (e.g. *F*, *t*, *r*) with confidence intervals, effect sizes, degrees of freedom and *P* value noted<br>*Give P values as exact values whenever suitable.*
☒	☐	For Bayesian analysis, information on the choice of priors and Markov chain Monte Carlo settings
☒	☐	For hierarchical and complex designs, identification of the appropriate level for tests and full reporting of outcomes
☐	☒	Estimates of effect sizes (e.g. Cohen's *d*, Pearson's *r*), indicating how they were calculated

Our web collection on statistics for biologists contains articles on many of the points above.

Software and code

Policy information about availability of computer code

Data collection	Confocal fluorescence microscopy images were acquired and analyzed using Leica TCS SP8 STED.
Data analysis	Mass spectrometry were then analyzed with an Orbitrap Classic mass spectrometry (Thermo Fisher Scientific Inc., MA, USA). Identification and quantification of the sample products were performed using MaxQuant version 1.5.0.30.The quantification for the proteins by western blot was performed by Image J version 1.51j8. All statistical analyses were performed using either GraphPad Prism 7 version 1.0 (GraphPad Software, Inc., La Jolla, CA, USA) or the Partek ® Genomics Suite ® version 7.0 (Partek Incorporated, St. Louis, MO, USA).

For manuscripts utilizing custom algorithms or software that are central to the research but not yet described in published literature, software must be made available to editors and reviewers. We strongly encourage code deposition in a community repository (e.g. GitHub). See the Nature Portfolio guidelines for submitting code & software for further information.

Data

Policy information about availability of data

All manuscripts must include a data availability statement. This statement should provide the following information, where applicable:

- Accession codes, unique identifiers, or web links for publicly available datasets
- A description of any restrictions on data availability
- For clinical datasets or third party data, please ensure that the statement adheres to our policy

The authors declare that all quantifications supporting the findings of this study are available within the paper, Source Data and the Supplementary Information files. Source data are provided with this paper. The RNA sequencing data generated in this study have been deposited in the GEO database under accession code: GSE182102 [https://www.ncbi.nlm.nih.gov/bioproject/?term=GSE182102]. The SILAC proteomics data of SRSF10 interactors generated in this study have been deposited in the PRIDE database under accession code: PXD030800 [https://www.ebi.ac.uk/pride/archive/projects/PXD030800/private]. The microarray data have

been deposited in the ArrayExpress public database under accession code: E-MEXP-84 [https://www.ebi.ac.uk/arrayexpress/experiments/E-MEXP-84/] and E-TABM-292 [https://www.ebi.ac.uk/arrayexpress/experiments/E-TABM-292/]. The SREK1L binding sites on the B-T mRNA were predicted by RNA-Protein Interaction Prediction (RPISeq) online database [http://pridb.gdcb.iastate.edu/RPISeq/about.php].

Field-specific reporting

Please select the one below that is the best fit for your research. If you are not sure, read the appropriate sections before making your selection.

☒ Life sciences ☐ Behavioural & social sciences ☐ Ecological, evolutionary & environmental sciences

For a reference copy of the document with all sections, see nature.com/documents/nr-reporting-summary-flat.pdf

Life sciences study design

All studies must disclose on these points even when the disclosure is negative.

Sample size	No statistical methods were used to pre-determine sample size. For animal works and RIP experiments, minimum sample sizes for individual experiments were determined not on the basis of a statistical method, but based on previous experience and report (Nat Commun . 2019 Mar 22;10(1):1353).
Data exclusions	no data were excluded
Replication	no experiments shown failed to replicated. All expeirments were replicated by at least three independent experments performed on different days.
Randomization	No specific randomization method was used to allocate mice into different treatment groups.
Blinding	Blinding was not necessary because experimental conditions were well-controlled and experimental results were quantitative and did not require subjective interpretation or analysis.

Reporting for specific materials, systems and methods

We require information from authors about some types of materials, experimental systems and methods used in many studies. Here, indicate whether each material, system or method listed is relevant to your study. If you are not sure if a list item applies to your research, read the appropriate section before selecting a response.

Materials & experimental systems

n/a	Involved in the study	
☐	☒	Antibodies
☐	☒	Eukaryotic cell lines
☒	☐	Palaeontology and archaeology
☐	☒	Animals and other organisms
☐	☒	Human research participants
☒	☐	Clinical data
☒	☐	Dual use research of concern

Methods

n/a	Involved in the study	
☒	☐	ChIP-seq
☒	☐	Flow cytometry
☒	☐	MRI-based neuroimaging

Antibodies

Antibodies used

The antibodies used in this studies were all listed in Supplementary table 5.
1) antibodies used for immunoblot:
Cat. No Antibody Lot No. Species dilution Company and description
HPA037674 SREK1 R35896 Rabbit 1/1000 Sigma-Aldrich, for Fig. 5G, Fig. 3E (ab-2)
#2118 GAPDH (14C10) 10 Rabbit 1/5000 Cell Signaling Technology
T6199 α-Tubulin (DM1A) R11234 Mouse 1/5000 Sigma-Aldrich
sc-393594 UPF1 L1217 Mouse 1/2000 Santa Cruz Biotechnology
sc-374557 SMG1 K2817 Mouse 1/1000 Santa Cruz Biotechnology
sc-515722 MOV10 H0916 Mouse 1/1000 Santa Cruz Biotechnology
sc-393905 RUVBL1 H0217 Mouse 1/1000 Santa Cruz Biotechnology
sc-374135 RUVBL2 K2817 Mouse 1/1000 Santa Cruz Biotechnology
sc-374230 UPF2 H1417 Mouse 1/1000 Santa Cruz Biotechnology
sc-398821 UPF3 G3117 Mouse 1/1000 Santa Cruz Biotechnology
sc-271405 MAGOH/B G0210 Mouse 1/1000 Santa Cruz Biotechnology
sc-514050 GNL2 I1217 Mouse 1/1000 Santa Cruz Biotechnology
sc-514308 SEC13 G2617 Mouse 1/1000 Santa Cruz Biotechnology
sc-32318 PABPC1 L2216 Mouse 1/1000 Santa Cruz Biotechnology
HPA053805 SRSF10 R71755 Rabbit 1/1000 Sigma-Aldrich

LS-C133661 SRSF10 NA Mouse 1/2000 LSBio
sc-33652 SRSF1 L0413 Rabbit 1/1000 Santa Cruz Biotechnology
HPA021593 PECR R09325 Mouse 1/1000 Santa Cruz Biotechnology
Sc-5261 ELAVL1/HuR NA Mouse 1/1000 Santa Cruz Biotechnology
Sc-101137 SFRQ NA Mouse 1/1000 Santa Cruz Biotechnology
HPA034829 SF3B6 R32766 Mouse 1/1000 Sigma-Aldrich
NA SREK1L NA Rabbit 1/5000 Customized made by Genscript (ab-1 of Fig 3E)
ab21679 CHC/CLTC GR264025-2 Rabbit 1/5000 Abcam
ab237697 TXNDC5 NA Rabbit 1/1000 Abcam
ab175205 BLOC1S5 NA Rabbit 1/2000 Abcam
A305105A SREK1L and SREK1S 1 Rabbit 1/5000 Thermofisher
sc-374015 Lamin B1 J2220 Mouse 1/5000 Santa Cruz Biotechnology

2) antibodies used for IP and SILAC:
Cat. No Antibody Lot No. Species Company Application
HPA037674 SREK1 R35896 Rabbit Sigma-Aldrich IP
A302-282A-1 SRSF10 NA Rabbit Bethyl Bio IP for SILAC
HPA053805 SRSF10 R71755 Rabbit Sigma-Aldrich IP for SILAC
NA SREK1-E10 NA Rabbit Customized, made by Genscript IP
I5006 IgG control NA Rabbit Sigma-Aldrich IP control
ab99617 Rat monoclonal [187.1] Secondary Antibody to Mouse kappa - light chain (HRP) GR1190308-3 Rat Abcam IB, 1/5000
ab99697 Mouse monoclonal [SB62a] Secondary Antibody to Rabbit IgG light chain (HRP) GR122803-1 Mouse Abcam IB, 1/5000

3) antibodies used for IF:
Cat. No Antibody Lot No. Species Company Dilution
ab89984 α-Tubulin GR271216-4 Chicken Abcam 1/1000
F9291 FLAG® BioM2/Flag-Biotin SLBF5390V Mouse Sigma-Aldrich 1/500
HPA037674 SREK1 R35896 Rabbit Sigma-Aldrich 1/200
LS-C133661 SRSF10 NA Mouse LSBio 1/100
ab5176 Anti-Histone H3 (phospho S10) NA Rabbit Abcam 1/200

Validation

All antibodies were validated by manufactures.
Anti-SREK1, https://www.sigmaaldrich.com/US/en/product/sigma/hpa037674
Anti-GAPDH, https://www.cellsignal.com/products/primary-antibodies/gapdh-14c10-rabbit-mab/2118
Anti-α-Tubulin, https://www.sigmaaldrich.com/US/en/product/sigma/t6199
Anti-UPF1, https://www.scbt.com/zh/p/rent1-antibody-c-6
Anti-SMG1, https://www.scbt.com/p/smg1-antibody-e-4
Anti-MOV10, https://www.scbt.com/p/mov10-antibody-b-3
Anti-RUVBL1, https://www.scbt.com/zh/p/pontin-52-antibody-a-11
Anti-RUVBL2, https://www.scbt.com/p/reptin-52-antibody-b-5
Anti-UPF2, https://www.scbt.com/p/rent2-antibody-g-10
Anti-UPF3, https://www.scbt.com/p/rent3-antibody-e-8/
Anti-MAGOH/B, https://www.scbt.com/p/magoh-antibody-c-11
Anti-GNL2, https://www.scbt.com/p/gnl2-antibody-b-8/
Anti-SEC13, https://www.scbt.com/p/sec13-antibody-f-6/
Anti-PABPC1, https://www.scbt.com/p/pabp-antibody-10e10/
Anti-SRSF10, https://www.sigmaaldrich.com/US/en/product/sigma/hpa053805
Anti-SRSF10, https://www.lsbio.com/antibodies/srsf10-antibody-fusip1-antibody-clone-1a6-elisa-if-immunofluorescence-wb-western-ls-c133661/137281
Anti-SRSF1, https://www.scbt.com/p/sf2-asf-antibody-96/
Anti-PECR, https://www.sigmaaldrich.com/US/en/product/sigma/hpa021593
Anti-ELAVL1/HuR, https://www.scbt.com/p/hur-antibody-3a2
Anti-SFRQ, https://www.scbt.com/p/psf-antibody-39-1
Anti-SF3B6, https://www.sigmaaldrich.com/US/en/product/SIGMA/HPA034829
Anti-CHC/CLTC, https://www.abcam.com/clathrin-heavy-chain-antibody-ab21679.html
Anti-TXNDC5, https://www.abcam.com/txndc5-antibody-ab237697.html
Anti-BLOC1S5, https://www.abcam.com/muted-antibody-epr11081b-ab175205
Anti-SREK1, https://www.thermofisher.cn/cn/zh/antibody/product/SREK1-SFRS12-Antibody-Polyclonal/A305-105A
Anti-Lamin B1, https://www.scbt.com/p/lamin-b1-antibody-b-10
Anti-SRSF10, https://www.bethyl.com/product/A302-282A/FUSIP1+Antibody
Anti-IgG, https://www.sigmaaldrich.com/US/en/product/sigma/i5006
Rat monoclonal Secondary Antibody, https://www.abcam.com/rat-monoclonal-1871-mouse-kappa-light-chain-hrp-ab99617.html
Mouse monoclonal [SB62a] Secondary Antibody, https://www.abcam.com/Mouse-monoclonal-SB62a-Rabbit-IgG-light-chain-HRP-ab99697.html
Anti-α-Tubulin, https://www.abcam.com/alpha-tubulin-antibody-loading-control-ab89984.html
Anti-Flag-Biotin, https://www.sigmaaldrich.com/US/en/product/sigma/f9291
Anti-Histone H3 (phospho S10), https://www.abcam.com/histone-h3-phospho-s10-antibody-ab5176.html

Eukaryotic cell lines

Policy information about cell lines

Cell line source(s)	The Hep3B (HB-8064), HepG2 (HB-8065), PLC/PRF/5(CRL-8024), SNU449 (CRL-2234), SK-Hep-1(HTB-52) and HEK-293T (CRL-3216) were obtained from American Tissue Culture (ATCC). Huh7 (JCRB0403), Huh1 (JCRB0199), HLE (JCRB0404), JHH2 (JCRB1028), JHH4 (JCRB0435), JHH5 (JCRB1029), JHH7 (JCRB1031) were obtained from Japanese Collection of Research Biosources Cell Bank (JCRB). SNU354 (KCLB00354), SNU368 (KCLB00368), SNU387 (KCLB00387), SNU398 (KCLB00398), SNU423 (KCLB00423), SNU449 (KCLB00449), SNU739 (KCLB00739), SNU761 (KCLB00761), SNU878 (KCLB00878), SNU886 (KCLB00886) cell lines were obtained from Korean Cell Line Bank (KCLB). The HCCLM3, Mahlavu and BEL7404 cells were gifts from Dr. John M Luk.
Authentication	Hep3B and HCCLM3 cells were examined by short tandem repeat analysis with the Geneprint 10 System Kit (Promega), the rest cell lines were not authenticated.
Mycoplasma contamination	all cell lines were tested negative for mycoplasma by every month monitoring.
Commonly misidentified lines (See ICLAC register)	No commonly misidentified cell lines were used.

Animals and other organisms

Policy information about studies involving animals; ARRIVE guidelines recommended for reporting animal research

Laboratory animals	5-7 week-old male BALB/c-Nude or C57BL/6 mice were purchased from INVIVOS PTE Ltd and housed in the animal facility at NCCS with the conditions: temperature range 19-26 °C, temperature fluctuations within bandwidth 3-4 °C, humidity range 50-70%, light intensity 300-350 lux at 1 meter above the floor, uniform 12 hours light/dark daily lighting cycle, ventilation 15-20 air change per hour.
Wild animals	no wild animals were used.
Field-collected samples	no filed-collected samples were used.
Ethics oversight	Mice were maintained according to protocols and ethical regualtions approved by SingHealth Institutional Animal Care and Use Committee (IACUC).

Note that full information on the approval of the study protocol must also be provided in the manuscript.

Human research participants

Policy information about studies involving human research participants

Population characteristics	The collection of tissues from HCC patients was approved by the SingHealth Centralized Institutional Review Board (CIRB), and all tissues studied were provided by the SingHealth Tissue Repository.
Recruitment	NA
Ethics oversight	SingHealth Centralized Institutional Review Board

Note that full information on the approval of the study protocol must also be provided in the manuscript.
